# Practical Notes and Formulæ

**Published:** 1886-02-20

**Authors:** 


### PRACTICAL NOTES AND FORMULÆ.

**Dr. Louries' Cholera Mixture.**—

℞. Chloroform.......................... ....min. xv–xx,
Tintura opii...............................min. x–xv,
Spiritus vini..............................fdr. i,
Aqua.......................................foz. j.

**Squibb's Cholera Mixture.**—

℞. Tinctura opii, }
Spirit camphoræ, } aa..........................foz. j,
Tinct. capsici, }
Chlorotormi purificat.........................fdr. iij,
Alcohol, 95%..................................q. s. ad. foz. v.

Dose, for persons over 18 years a teaspoonful.

**Ruschenberger's Cholera Mixture.**—

℞. Tinct. zingiberi, }

Tinct. capsici, }

Tinct. piperitæ, } aa.....................equal parts.

Tinct. opii. }

**Digestive Wine.**—Take of—

℞. Pepsin.......................................24 grs.
Glycerin......................................2 fl. ozs.
Tinture of columbo...........................4 fl. drs.
White wine................. ..............q. s.

Dissolve the pepsin in the glycerin ; add successively about six fluid ounces of good maderia or dry sherry, free from excess of acid ; pour the mixture into a clean, dry wine bottle ; fill it up with the same wine, cork well, and let it remain at least one month, lying on its side, to mature. Any ordinary wineglassful to be taken with dinner, or two glasses, if such has been the previous habit of the patient. A fine tonic, stomachic, and digestive.

**Good Mucilage.**—(Therapeutic Gazette) A mucilage of acacia, which will not spoil, may be made according to the following formula :

℞. Oil of gaultheria..........................min. xv,

Calcium phosphate.........................q. s.,

Water.......................................ʒ viii,

Acacia......................................ʒ iv.

**Chronic Nasal Catarrh.**—(New England Medical Monthly) Prof. Agnew says he has cured cases of chronic catarrh accompanied by profuse discharges by douches of sage tea. Other remedies had been tried in vain.

**Prescription for Apthous Sore Mouth.**—(Southern Clinic) For apthous mouths of infants Bienert recommends in the Rundschau Leitmeritz the following: Distilled glycerin, 1000 parts; pulverized borax, 50 parts; spirits of rose, (1:100) 1 to 2 parts; catechu, 10 parts; tannic acid, 2 parts. Mix and use with a swab.

**For Constipation in Young Children.**—(Southern Clinic) Dr. Poulain, in British Medical Journal, extols the use of a tablespoonful of fine bran night and morning, in a cup of bread and milk. The bran is warmed in the milk and then poured on the bread.

**To Disguise the Taste of Quinine.**—(Southern Clinic) Dr. Line recommends the following formula:

℞. Ext. yerba santa fld.............................℥ j,

Syr. simple.............................................℥ iij,

Quinia sulph...........................................grs. xxx.

M. Dose, a teaspoonful.

**Chronic Rheumatism.**—(Medical Bulletin)

℞. Potassii iodidi.........................................ʒ iss,

Ammonii chloridi.......................................ʒ j,

Spts. chloroformii......................................ʒ ss,

Tinc. cinchonæ com...................................℥ iv.

M. S. Two teaspoonfuls three times a day.

**For Painful Hemorrhoids.**—(Dr. U. I. Niles, in N. E. Medical Monthly) This ointment has been a favorite of mine for several years, and I send it to the Clinic for the use of your readers:

℞. Extract of hyosciamus, } aa..................ʒ ijss,

Pulv. saffron, }

Plumbi acetas...........................................ʒ j,

Glycerole of Starch...................................℥ j.

M.

**Anodyne for Children.**—(Dr. R. St. J. Perry, Indiana Pharmacist) The following "Baby ℞" is frequently used in the City Hospital, and has generally given much satisfaction:

℞. Sodii brom..............................4 scruples,
Oil anisi.....................................2 grains,
Tr. opii camph...........................32 drops,
Aqua, q. s., ad............................2 ounces.

M. Sig. Teaspoonful every hour, as needed. Shake before using.

**Chronic Diarrhœa.**—A writer in Eclectic Medical Journal recommends the following superior remedy: ℞. Distillate Hamamelis, water, aa f. ℥ ij; bin-iodide hydrarg, gr. j. M. S.: Dose half teaspoonful every three hours. Mercurial salt to be dissolved in two fluid drachms of alcohol before mixing with the watery vehicle.

